# Characteristics of 263K Scrapie Agent in Multiple Hamster Species

**DOI:** 10.3201/eid1502.081173

**Published:** 2009-02

**Authors:** Kimberly D. Meade-White, Kent D. Barbian, Brent Race, Cynthia Favara, Don Gardner, Lara Taubner, Stephen Porcella, Richard Race

**Affiliations:** National Institute of Allergy and Infectious Diseases, Hamilton, Montana, USA

**Keywords:** Transmissible spongiform encephalopathy, TSE, species barriers, molecular profile, biochemical profile, PRNP amino acid sequence, prnp nucleotide sequence, hamsters, scrapie, research

## Abstract

Transmissible spongiform encephalopathy (TSE) diseases are known to cross species barriers, but the pathologic and biochemical changes that occur during transmission are not well understood. To better understand these changes, we infected 6 hamster species with 263K hamster scrapie strain and, after each of 3 successive passages in the new species, analyzed abnormal proteinase K (PK)–resistant prion protein (PrPres) glycoform ratios, PrPres PK sensitivity, incubation periods, and lesion profiles. Unique 263K molecular and biochemical profiles evolved in each of the infected hamster species. Characteristics of 263K in the new hamster species seemed to correlate best with host factors rather than agent strain. Furthermore, 2 polymorphic regions of the prion protein amino acid sequence correlated with profile differences in these TSE-infected hamster species.

## Abstract 2

Characteristics correlated better with host factors than with agent strain.

Transmissible spongiform encephalopathy (TSE) diseases are infectious, fatal, neurodegenerative diseases of the central nervous system that affect a wide variety of mammals, including humans. In the past several decades, 3 new TSE diseases have been identified in different species: chronic wasting disease of deer and elk, bovine spongiform encephalopathy of domestic cattle, and variant Creutzfeldt-Jakob disease of humans. Thus, a better understanding of the process of cross-species transmission is needed.

Recognition of natural cross-species transmission is not straightforward. If a disease that crosses species has clinical or pathologic features similar to those of an already well-characterized TSE disease, it may not be recognized as a cross-species infection. Furthermore, some cross-species events involve slow processes in which the TSE agent adapts over several passages before recognizable clinical disease occurs (*1*).

The amino acid sequence of the prion protein (PrP) is known to be an influential factor for cross-species transmission of TSE disease to a new host. Multiple single nucleotide polymorphisms resulting in amino acid changes that control susceptibility to TSE disease have been identified in sheep, cervids, humans, and transgenic mice (*2*–*6*). Similarly, incubation periods have also been correlated with nonsynonomous single nucleotide polymorphisms in the PrP gene. For example, 2 polymorphic residues at amino acids 108 and 189 are associated with either short (Leu108/Thr189) or long (Phe108/Val189) incubation periods in mice (*7*). Even a single point mutation in mice at amino acid position 101 has been shown to alter proteinase K–resistant prion protein (PrPres) deposition in brain, incubation periods, and host range (*8*).

In this study, we examined molecular and biochemical changes associated with cross-species transmission in 6 hamster species of the rodent subfamily *Cricetinae.* All animals were handled according to the National Institutes of Health guidelines and protocols approved by the Rocky Mountain Laboratories’ (Hamilton, MT, USA) Institutional Animal Care and Use Committee**.**

Phylogenetic classification of these hamster subspecies is based on DNA sequences of mitochondrial cytochrome *b* gene and a portion of the NADH dehydrogenase 4 gene (*9*). These 6 species diverge into 3 genera, mainly *Cricetulus* (including Armenian and Chinese), *Phodopus* (including Djungarian and Siberian), and *Mesocricetus* (including Turkish and Syrian) hamster species (Figure 1). By inoculating each of these hamster species with a well-characterized, stable strain of Syrian hamster scrapie (263K), we were able to compare and analyze molecular and biochemical parameters of cross-species transmission events. To identify the cross-species transmission event, we looked for recognizable features that may have emerged in the new host. We found that each new host species presented a profile unlike that of the original Syrian hamster host infected with 263K. These profile changes correlated with unique PrP amino acid sequences within the 6 hamster species. This finding suggests that host PrP sequences can change the phenotype presentation of the agent in the host and could thereby confound identification of cross-species transmission events.

**Figure 1:**
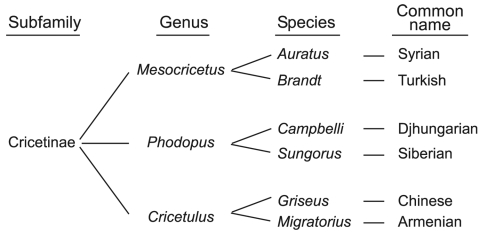
Taxonomic classification for 6 hamster species. Phylogenetically, these species are grouped into closely related taxonomic genera (*9*).

## Materials and Methods

### Incubation Periods and Titers for Passaged 263K

The original source of 263K hamster agent came from Kimberlin et al. (*10*) and was passaged 3 times in Syrian hamsters at Rocky Mountain Laboratories. For cross-species transmissions, also referred to as first passage, weanling hamsters from each of the 6 hamster species were intracranially inoculated with 263K stock at a titer of 2 × 10^9^ lethal dose for 50% per 50 µL of a 1% brain homogenate. Clinically ill hamsters from each species were killed, and 1% brain homogenate was passaged intracranially into weanling hamster recipients of the same species (second passage). The process was repeated for a third passage. Brain homogenate from clinically ill third-passage hamsters was inoculated intracranially back into Syrian (back-passaged) hamsters.

**Figure 2:**
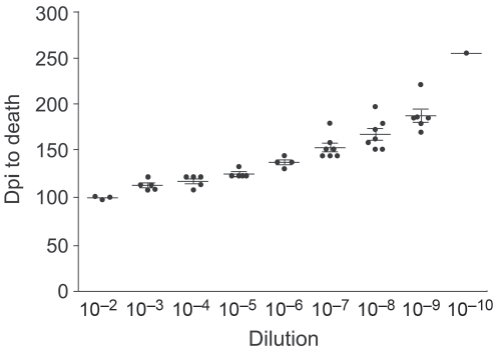
Example of titration curves for all homogenates titered. The curve shown is Djungarian second-passage brain homogenate with 3–7 hamsters per dilution. Error bars indicate SEM. Dpi, days postinoculation.

Each hamster was killed when it had lost ≈30% of its body weight and was no longer able to remain upright and feed itself. We determined endpoint titrations for 263K, first, second, and third hamster passage inocula used in these experiments for all species (except Chinese hamsters) by preparing sequential 1:10 dilutions of a 1% brain homogenate to 10^–8^ or 10^–10^. Dilutions were then injected intracranially into 6–8 hamsters for each dilution, and titers were determined as described (Figure 2) (*11*).

### Immunoblot Analysis of Proteinase K–Sensitive Prion Protein and PrPres by Western blot

Proteinase K (PK)–sensitive prion protein (PrPsen) and PrPres were prepared as previously described (*12*,*13*). Samples were frozen at –20º C until they were subjected to electrophoresis on a 16% sodium dodecyl sulfate–polyacrylamide gel (Invitrogen, Carlsbad, CA, USA). Immunoblots were probed by using polyclonal antibody R30 to PrP (89–103 in Syrian hamsters) (*14*,*15*), which recognizes PrP from each of the 6 species. Blots were developed by using either enhanced chemiluminescence or enhanced chemifluorescence according to manufacturer’s instructions (Amersham-Pharmacia, Uppsala, Sweden). Enhanced chemifluorescence blots were scanned by using a STORM fluorescent detection system (Amersham-Pharmacia) as described previously (*16*).

### Sensitivity of Proteinase K

 To demonstrate PrPres sensitivity to PK, we adjusted 20 μL of a 20% (wt/vol) third-passage brain homogenate in 0.01 M Tris, pH 7.3, from each species to 100 mmol/L Tris HCl, pH 8.3, 1% Triton X-100, and 1% sodium deoxycholate. Samples were treated with 25 μg/mL, 100 μg/mL, 400 μg/mL, or 1,600 μg/mL PK in a total volume of 35 μL and incubated at 37º for 1 h. The reaction was stopped by adding 2 μL of 0.1 M phenylmethylsulfonyl fluoride and placed on ice for 10 min. Samples were then mixed in equal volumes with 2× sample buffer, boiled 5 min, and subjected to electrophoresis on sodium dodecyl sulfate–polyacrylamide gels. PrP bands were quantitated as described above.

### PrP Gene Sequencing

To sequence the PrP gene open reading frame (ORF), we based primers on published regions of sequence homology between the Syrian, Armenian, Chinese hamster; rat; and mouse PrP genes. Primers 7F, S/O(–1), C(–1), S/T(+2), C/O(+2), and 1R are located outside the ORF. The following primers were used: 7F-5′-GCCTTGTTCTTCATTTTGCAGA-3′, S/O(–1) 5′-TCATTTTGCAGATCAGCCATC-3′, C(–1) 5′-TCATTTTGCAGATTAGCCAT-3′, S/T(+2) 5′-GTACAAGCAGGGAGGCTTCCTTC-3′, C/O(+2) 5′-GTACAAGCAGGGAGGCTTCCCTC-3′, and 1R 5′-ACCCCTCCCCCAGCCTAG-3′.

### Immunohistochemistry

Immunohistochemistry procedures were conducted as previously described (*12*). PrPres was detected by using R30 anti-PrP antibody (residues 89–103).

## Results

### Incubation Periods and Titers

Incubation periods and infectivity titers are among the criteria used to define TSE strains (*17*,*18*). Therefore, we compared incubation periods and infectivity titers for 263K in Syrian hamsters with those observed for the 5 other hamster species. Cross-species transmission of Syrian-derived 263K to Turkish hamsters, both members of the genus *Mesocricetus* (Figure 1), had similar incubation periods (Table). Transmission of 263K to hamsters of the genus *Phodopus* (Djungarian and Siberian) had incubation periods similar to each other but different from those of the *Mesocricetus* hamsters. Although Chinese and Armenian hamsters each belong to the genus *Cricetulus*, after inoculation with 263K, their incubation periods differed from each other and from those of all the other species. In most instances, we observed prolonged incubation periods when Syrian 263K was inoculated into the new hosts. Because all the hamsters received the same inoculum at passage 1, this finding likely reflects the species barrier between the hosts (*19*) rather than inoculum titer.

**Table:** Average incubation period for 263K scrapie*

Hamster species†	Cross-species 263K			Second passage			Third passage			Back passage‡
	Dpi ± SD	Titer/50 μL (1% BH)		Dpi	Titer/50 μL (1% BH)		Dpi	Titer/50 μL (1% BH)		Dpi
Syrian	85	2 x 10^9^		ND	ND		ND	ND		79 ± 5
Turkish	91 ± 4.9	1 x 10^8.9^		103 ± 7.2	1 x 10^6.7^		85 ± 7.6	1 x 10^8.25^		76 ± 0
Djungarian	155 ± 23.4	1 x 10^8.4^		100 ± 9.0	1 x 10^9.6^		99 ± 7.2	1 x 10^9.7^		97 ± 7
Siberian	148 ± 26.5	1 x 10^8.3^		117 ± 9.9	1 x10^7.75^		114 ± 8.3	1 x 10^8.1^		128 ± 17
Chinese	372 ± 29.1	ND		233 ± 17.9	ND		207 ± 15.2	ND		168 ± 7
Armenian	188 ± 10.4	1 x 10^7.35^		156 ±4.8	1 x 10^7.75^		145 ±7.5	>1 x 10^8.5^		145 ± 18

*Dpi, days postinoculation; ND, not done; BH, brain homogenate. †16 hamsters/species. ‡In Syrian hamster.

Two additional passages from a donor to a recipient of the same species showed that incubation periods in most species differed considerably from those of the original Syrian hamsters (Table). Incubation periods for the new species decreased and then became stable between second and third passage and were considerably different from those of the original Syrian hamsters (Table). Incubation periods for Chinese hamsters were still decreasing between second and third passages. Because a fourth passage was not performed for Chinese hamsters, whether third passage reflects the stable incubation period is unknown. Thus, for Djungarian, Siberian, and Armenian hamsters, evidence was strong for unique incubation periods in the new species by second passage after inoculation with Syrian 263K agent.

To determine whether incubation period differences for each species resulted from differing infectivity concentration in the inocula, we used endpoint titration to determine the brain titers after first, second, and third passages (Table). These determinations were not conducted for Chinese hamsters because the long incubation periods would require several additional years. When comparing first-passage titers with titers in Syrian hamsters infected with 263K, the only decrease in infectivity titer was seen in Armenian hamsters (Table). When comparing second-passage titers with titers in Syrian hamsters infected with 263K, we observed a minimal decrease in Turkish, Siberian, and Armenian hamsters. We observed no notable changes in titers among third passage animals of all 6 species, which suggests stable titers in each species by third passage.

Additional evidence for adaptation of 263K agent to the new host species was obtained by infecting Syrian hamsters with brain homogenates from third-passage hamsters. If the agent had adapted to the new species, we would expect to see differences in incubation periods. In contrast, in the absence of adaptation, we would expect reversion back to the characteristic 263K Syrian incubation period. The only hamster for which third-passage brain homogenate resulted in a decrease in incubation period was the Chinese hamster. Even so, at 168 days this incubation period still differed from the characteristic 80-day incubation period for Syrian hamsters. These results indicate that the 263K agent adapted to the new host and that each 263K-infected hamster species has a unique incubation period (Table).

### Glycoform Profiles

PrPres glycoform profiles are another criteria used to differentiate TSE strains (*20**–**22*). Therefore, we compared PrPres glycoform profiles from each of the 6 hamster species at each passage.

**Figure 3:**
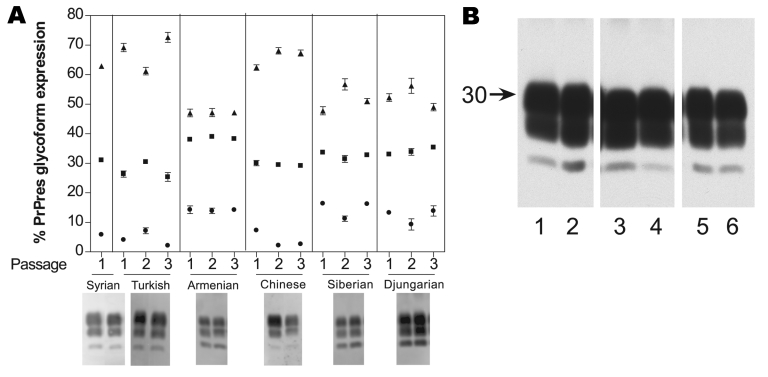
A) Proteinase K–resistant prion protein (PrPres) glycoform profiles for 6 hamster species for each of 3 successive passages: 1, initial cross-species passage; 2, second intraspecies passage; 3, third intraspecies passage. Each passage represents 6 different animals, each quantified 6–8 times. Each lane had 0.5 mg tissue equivalents per lane. ●, percentage of unglycosylated band; ■, partially glycosylated band; ▲, fully glycoslyated band. Western blot representation of glycoform for each species visualized using R30 and enhanced chemifluorescence. Error bars indicate SEM. B) Serially passaged 263K scrapie from 5 hamster species passaged back to Syrian hamster. Western blot analysis of clinically ill Syrian hamster infected with Syrian 263K or 263K passaged 3 times through the new hamster host. Syrian hamster inoculated with brain homogenate from the following hamsters: lane 1, Syrian 263K; 2, Turkish; 3, Chinese; 4, Armenian; 5, Djungarian; 6, Siberian. Tissue equivalents: lane 1, 0.5 mg; lanes 2, 5, and 6, 0.4 mg; lane 3, 0.7 mg; and lane 4, 0.9 mg.

When Western blotting was used to compare percentages between the 3 PrPres bands (Figure 3, panel A), the data clearly showed 2 different PrPres glycoform profiles. The Turkish hamster PrPres glycoform profile shared similarities with that of both Syrian and Chinese hamsters. Siberian, Djungarian, and Armenian hamsters shared a second PrPres glycoform profile. PrPres glycoform patterns from Turkish, Chinese, Siberian, and Djungarian hamsters fluctuated noticeably over the 3 passages, which suggests a lack of stability while adapting to the new species. In contrast, PrPres glycoform patterns from Armenian hamsters did not fluctuate over the 3 passages, which suggests a stable strain in Armenian hamsters (Figure 3, panel A).

We also injected brain homogenates derived from the third-passage hamsters back into Syrian hosts. We found that glycoform patterns in the Syrian recipients were the same as those ordinarily associated with Syrian hamsters (Figure 3, panel B), which suggests, as with incubation periods, that the host had a predominant influence over glycoform patterns.

### PrPsen

**Figure 4:**
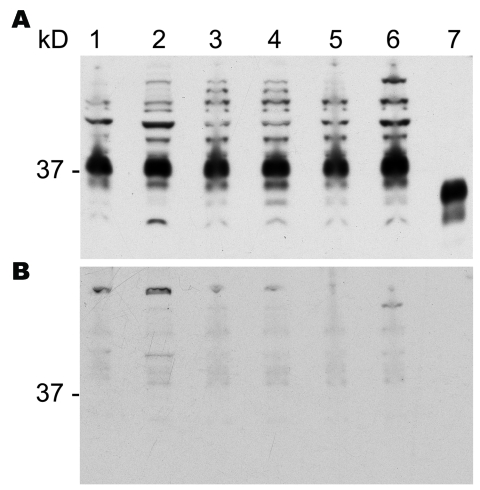
Proteinase K–sensitive prion protein (PrPsen) Western blot analysis from 6 hamster species performed with A) polyclonal antibody R30 (89–103) or B) R30 preincubated with peptide to prion protein 89–103. Hamster species: lane 1, Syrian; lane 2, Turkish; lane 3, Djungarian; lane 4, Syrian; lane 5, Chinese; lane 6, Armenian. Lane 7, proteinase K–resistant prion protein (PrPres) from 263K Syrian hamsters. 0.8 mg tissue equivalents per lane; 37 kDa indicated.

To investigate the possibility that differences in PrPres glycoform profiles were reflections of different PrPsen characteristics in the various hamster species, we analyzed PrPsen profiles by using Western blot. No differences were found in expression levels or banding patterns among the 6 hamster species (Figure 4, panel A). Bands detected at 37 kDa were proven to be PrPsen because they were competed out (protein signal disappeared) when we preincubated the antibody to PrP with a synthetic peptide specific for the PrP epitope. Bands >37 kDa did not compete out (Figure 4, panel B). All PrPsen samples were PK sensitive (data not shown).

### PK Resistance

PrPres resistance to PK digestion has also been used to differentiate TSE strains (*23*–*25*). When we compared PrPres resistance to PK from 263K Syrian and third-passage Turkish, Armenian, Chinese, Siberian, and Djungarian hamsters, we found no differences in sensitivity to PK. All samples retained equivalent PrPres signals on Western blots after treatment with 25 and up to 1,600 μg/mL PK (data not shown).

### Immunohistochemical Findings

**Figure 5:**
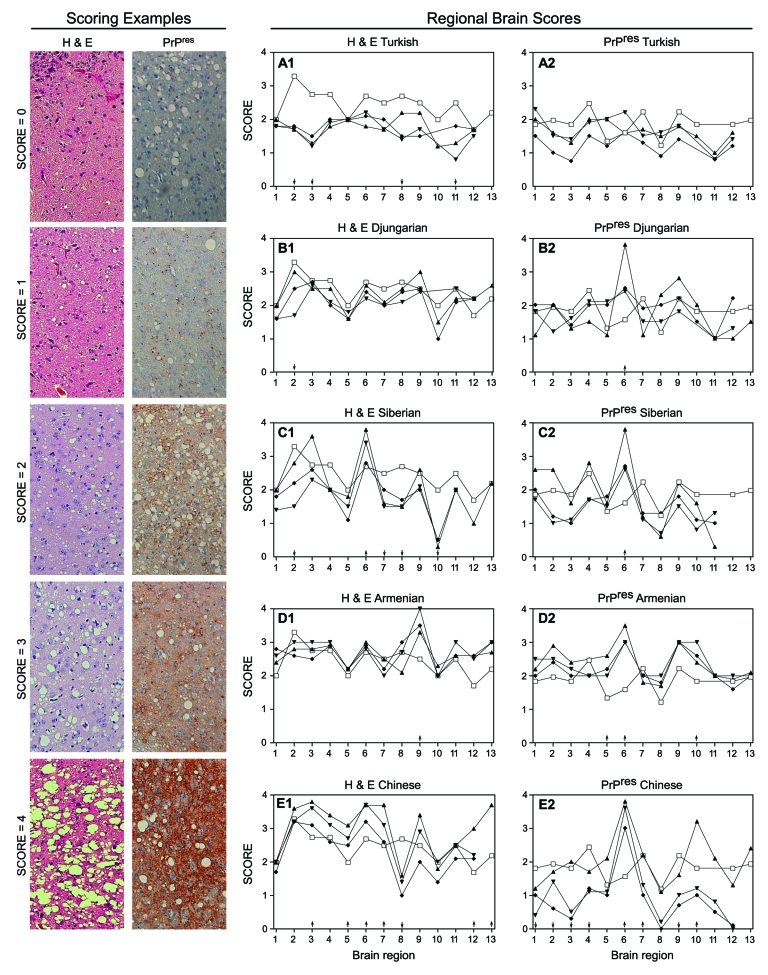
Scoring examples: Hematoxylin and eosin (H&E)–stained and proteinase K–resistant prion protein (PrPres) scores from 0 to 4. A score of 0 means no vacuolation or PrPres distribution; a score of 4 means the highest degree of vacuolation and PrPres distribution in that region. Panel H&E score: 0, Chinese hamster olfactory bulb; 1, Syrian hamster spinal cord; 2, Armenian hamster caudate putamen; 3, Armenian hamster cortex; 4, Syrian hamster thalamus. Panel PrPres score: 0, Chinese hamster superior colliculus; 1, Syrian hamster posterior colliculus; 2, Chinese hamster brain stem; 3, Djungarian hamster posterior colliculus; 4, Chinese hamster thalamus. Regional Brain scores: all hamsters were compared with 263K Syrian hamster (□) shown in each panel A1–E1 and A2–E2. Lesion and PrPres profiles of 263K scrapie-infected hamster from each of the 5 new hamster species. Each point represents the average from 6 different animals scored in the following areas: 1, cerebellum; 2, posterior colliculus; 3, superior colliculus; 4, brain stem; 5, spinal cord; 6, thalamus; 7, hypothalamus; 8, hippocampus; 9, cortex; 10, olfactory bulbs; 11, caudate putamen; 12, septal nucleus; 13, tegmentum. ▲, first passage; ▼, second passage; ♦, third passage.

Differentiation of TSE strains has also been based on regional distribution of PrPres and microscopic lesions in brains of infected individuals (*18*). Therefore, we studied lesion profiles in brain from 5–8 hamsters of each species at each passage. We scored 13 areas from 0 to 4 (none to the highest degree of PrPres distribution or lesions) (Figure 5, Scoring Examples). Averages for each brain region were scored and compared (Figure 5, Regional Brain Scores). A score change >1 between species or passages was considered a notable change for that region (*26*).

Microscopic lesion profiles (Figure 5, panels A1–E1) from the 3 passages for each species were compared with those of the 263K Syrian hamsters (stained with hemotoxylin and eosin [H&E]). We found that Djungarian hamster profiles were most similar to 263K Syrian hamster profiles. Second-passage Armenian hamsters had increased vacuolation in the cortex; Turkish, Siberian, and Chinese hamsters differed in multiple regions. Lesion profiles for the 3 passages in each of the new hamster host species, such as Chinese hamsters (Figure 5, H&E Chinese) regions 2 and 3, did not necessarily correlate with PrPres distribution (Figure 5, panel PrPres Chinese) within that host.

When comparing the PrPres deposition profile (Figure 5, PrPres panels [right side]) at each of the 3 passages to the Syrian PrPres deposition profile, we found that the profiles of the Turkish hamsters were the most similar. Because Syrian and Turkish hamsters are closely related phylogenetically and share similar PrPres glycoform patterns, this finding was not surprising. In the other 4 species (Djungarian, Siberian, Armenian, and Chinese), PrPres distribution in the thalamus was increased (region 6) over that in the same region for Syrian hamsters. Further changes were seen in Armenian hamsters; PrPres distribution was increased in the spinal cord (region 5) and olfactory bulb (region 10). PrPres distribution patterns in Chinese hamsters diverged from those in the Syrian hamsters in almost every region except the spinal cord (region 5). In all 6 species, including our original Syrian hamster species, unique pathologic phenotypes developed. Immunohistochemical and glycoform profiles for 2 of the 3 genera were independently unique, which suggests that similar host factors within those genera influenced these TSE characteristics.

**Figure 6:**
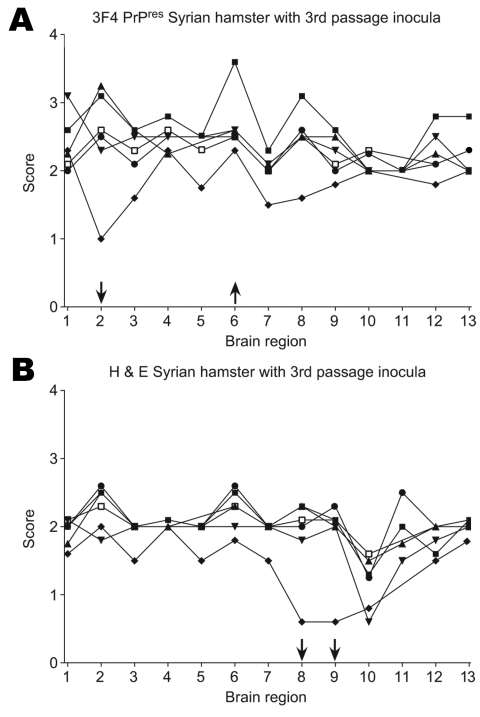
A) Proteinase K–resistant prion protein (PrPres) pathogenicity profiles in Syrian hamsters inoculated with third-passage PrPres. B) Hematoxylin and eosin (H&E)–stained lesion profiles of Syrian hamsters inoculated with brain homogenate derived from third-passage hamsters. Each point represents the average from 6 different animals scored in the following areas: 1, cerebellum; 2, posterior colliculus; 3, superior colliculus; 4, brain stem; 5, spinal cord; 6, thalamus; 7, hypothalamus; 8, hippocampus; 9, cortex; 10, olfactory bulbs; 11, caudate putamen; 12, septal nucleus; 13, tegmentum. Syrian hamster inoculated with third-passage brain homogenate from the following hamster species: ●, Turkish; ■, Djungarian; ♦, Chinese; ▼, Armenian; ▲, Siberian; □, Syrian. Arrows represent differences in multiple regions indicating increases or decreases in vacuolation or PrPres deposition.

To determine the respective contributions of the agent and host in immunohistochemical patterns, we injected brain homogenate from third-passage hamsters back into Syrian hamsters. When patterns were compared with those of 263K Syrian hamster, only a few differences from 263K Syrian were observed. Specifically, less PrPres was deposited in the posterior colliculus (region 2) in Syrian hamsters inoculated with brain homogenate from Chinese hamsters, and more PrPres was deposited in the thalamus (region 6) of Syrian hamsters inoculated with brain homogenate from Djungarian hamsters (Figure 6, panel A). We also observed a decrease in vacuolation in the hippocampus (region 8) and cortex (region 9) of Syrian hamsters inoculated with brain homogenate from Chinese hamsters (Figure 6, panel B). For all other hamster species inocula, no remarkable differences from 263K Syrian hamster were noted. Therefore, the host appeared to play an important role in determining immunohistochemical patterns. It is not known whether the differences seen in the Chinese and Djungarian hamsters are due to donor species effect (*27*,*28*), whereby a temporary change occurs as an agent is passed through a new host species, or whether selection of a new strain has occurred. To answer this question, a second passage through Syrian hamsters is needed. These results suggest that although 263K agent was substantially altered by passage through the new hamster species, in general it was not able to impart molecular characteristics like glycoform or the pathologic patterns established in the new species back to the Syrian hamsters.

### Sequencing

Different PrP amino acid sequences have been associated with variation in lesion profiles, incubation periods, and susceptibility to TSE disease in humans and in sheep, cervid, and mouse models. We report the PrP gene sequences for Siberian, Djungarian, and Turkish hamsters. To determine how amino acid sequence might associate with the TSE characteristics we observed here, we compared our new sequences with the published PrP gene ORF of Syrian (*29*), Chinese, and Armenian hamsters (*30*,*31*).

**Figure 7:**
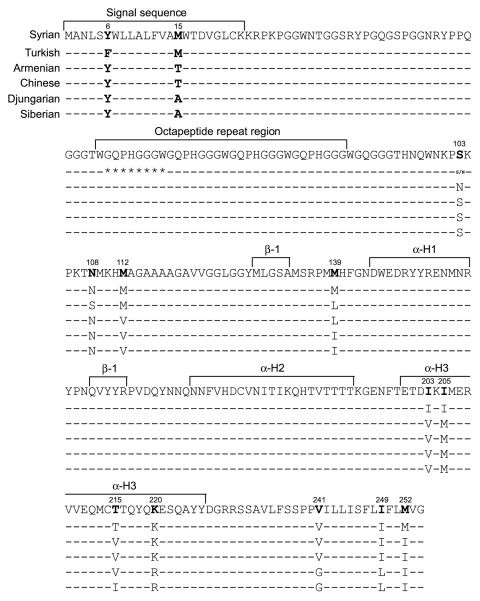
Sequences of prion protein for 3 hamster species. Hamster genomic DNA was purified from whole uninfected hamster brain tissue by using the QIAamp DNA blood maxi 10 kit columns and the solutions and tissue protocol from QIAGEN Dneasy Blood and Tissue kit (QIAGEN, Valencia, CA, USA). PCR products were amplified by using both PuReTaq Ready-To-Go PCR beads (GE Healthcare, Piscataway, NJ, USA) and Expand High-Fidelity Taq polymerase (Roche Diagnostic Corp, Indianapolis, IN, USA). Successful amplicons were purified by using QIAquick PCR Purification kit (QIAGEN) according to manufacturer’s recommendations and sequenced by using their respective forward and reverse PCR primers with an ABI 3730xl DNA Analyzer (Applied Biosystems, Foster City, CA, USA). Sequence data were stored in the FINCH data management system (Geospiza, Seattle, WA, USA). Assembly and comparisons were made against the Syrian prion protein gene sequence by using Sequencher (Gene Codes, Ann Arbor, MI, USA). Amino acid substitutions are indicated; otherwise, sequence matches that of Syrian hamster.

When comparing the new *prnp* sequences to the Syrian *prnp* sequence, we found considerable variation in nucleotides. Siberian hamsters had 40 nt changes, and Djungarian hamsters had 41. These nucleotide changes resulted in 10 aa substitutions for both species; only 1 aa acid substitution, I215V, distinguished sequences between Siberian from Djungarian hamsters (Figure 7). Only Turkish and Syrian hamsters had 3 nt differences, resulting in 2 aa substitutions. An amino acid Y-to-F substitution was found at codon 6 in the signal sequence, and a heterozygous base was found at codon 103 (encoding both S and N). In addition, Turkish hamsters have a deletion of 1 octapeptide repeat (Δ81–87) on both alleles, a characteristic shared with African Green monkeys (*32*). In humans, deletion of 1 octapeptide repeat occurs at a frequency of 0.5% and is not associated with disease (*33*,*34*). We found 34 nt changes for Armenian and 37 for Chinese hamsters, resulting in 7 aa substitutions for each hamster species, a finding that agrees with published data (*30*). The 3 aa differences that distinguish Armenian from Chinese hamsters are located at aa positions 103, 108, and 112 (*30*).

## Discussion

Of the 6 hamster species inoculated with a well-defined hamster scrapie source, each species not only was susceptible to the agent but also developed unique PrPres biochemical and pathologic characteristics. These characteristics, which included incubation periods, PrPres glycoform patterns, and PrPres distribution in brain, appeared to segregate into related genera with the exception of the genus *Cricetulus* (Armenian and Chinese hamsters). Because TSE characteristics were unique for 2 of 3 genera, a role for host factor involvement is implicated. Our data suggest that PrP amino acid sequence is the host factor responsible for the unique characteristics.

Data from our study and another experimental study (*30*) suggest that not only is the host responsible for determining TSE characteristics such as incubation periods but so is the agent. Our data show that closely related hamsters infected with the same strain of TSE have different incubation periods. These differences cannot be explained by differential expression levels of PrPsen because by Western blot, PrPsen expression was equivalent (Figure 4, panel A). Also, we believe that changes in incubation periods cannot be attributed to differential infectivity titers because only minor variations in titers were found. A second set of experiments showed that the same hamster host inoculated with different strains of TSE had different incubation periods, such as hyper (65 ± 1) and drowsy (168 ± 2) (*23*).

Because incubation periods and altered PrPres deposition in the brain have been linked to polymorphisms in the PrP amino acid sequence (*8*,*35*), we investigated the possibility that PrPres biochemical and pathologic changes correlate with different host PrP amino acid sequences. When comparing the PrP gene ORF for each of the 6 hamster species, we found 13 possible amino acid substitutions localized to 2 regions, either the N or the C terminus. Hamsters from 2 genera in this study (*Phodopus* and *Mesocricetus*) maintained sequence homogeneity at these 13 residues and had very similar TSE characteristics. Hamsters of the third genus, *Cricetulus,* differed in PrP gene sequence at 3 amino acids within the N-terminal polymorphic region. These polymorphic changes could explain why Armenian and Chinese hamsters have such different incubation periods, PrPres glycoform patterns, and immunohistochemical profiles from each other as well as from the 2 genera *Cricetulus* and *Phodopus.* It is also possible that closely related hamsters have unidentified genes that also influence TSE characteristics.

Our results are consistent with the published data indicating that amino acids 102–139 may control scrapie incubation periods in experimental models (*7*,*30*,*36*). In addition, our data suggest that PrPres glycoform profiles and lesion profiles are also associated with this region. Armenian and Chinese hamsters, both in the genus *Cricetulus,* show significant differences from each other, having only 3 aa substitutions at residues 103, 108, and 112 in this N-terminal polymorphic region. In contrast, amino acid sequences from Djungarian and Siberian hamsters were the same in this region, and those from Turkish hamsters matched those from the Syrian hamsters. These findings suggest a direct role for these residues and the N-terminus in determining TSE characteristics. In addition, all 3 genera differ from each other at residue 139, the same position thought to participate in the hydrophobic core potentially stabilizing PrPsen in mice (*37*). The unstructured nature of this region has led some investigators to consider this stretch of residues as a potential nucleation site for conversion of PrPsen to PrPres (*37*). Sequence differences in this position may dramatically affect initial conversion events.

Certain amino acid substitutions may play only a minor role in TSE strain profiles. For instance, residue V215T is thought to be responsible for the structural differences between mouse and hamster PrPsen by introducing a bend in helix 3 (*38*). From our data, T215V/I substitutions may have had a minor in vivo effect with respect to TSE characteristics. Djungarian and Siberian hamsters differed only at this amino acid and had similar glycoform and IHC patterns as well as incubation periods.

Amino acid substitutions identified in this study segregated into genera with similar incubation periods, glycoform profiles, and brain pathologic changes. Because we used only 1 TSE agent, these changes can be attributed to the host factors and not to the agent. A previous study of 3 hamster species also concluded that host rather than agent had a predominant role in determining biochemical and molecular PrPres attributes (*30*). Because host factors seem to play a larger role in determining the TSE profile, identifying the source of infection in cross-species infections may be difficult, which would be especially worrisome when species important to humans (e.g., sheep, cattle, cervids) are involved. These ruminant species are heterogeneous and not nearly as closely related as the hamsters studied here.

Our study links PrP amino acid sequences to biochemical and pathologic profiles of PrPres in multiple hamster species infected with 263K hamster scrapie. To further investigate how specific amino acid substitutions in PrP are associated with specific TSE characteristics, these hamster and similar PrP gene sequences could be expressed in tissue culture assays and protein modeling experiments. The results of these experiments could broaden our understanding of TSE profiles and the role that PrP amino acid residues play, possibly leading to assays that could identify instances of cross-species transmission.
